# Response to: Comment on “Role of Mitochondrial Genome Mutations in Pathogenesis of Carotid Atherosclerosis”

**DOI:** 10.1155/2018/7620234

**Published:** 2018-08-09

**Authors:** Igor A. Sobenin, Margarita A. Sazonova, Vasily V. Sinyov, Anastasia I. Ryzhkova, Elena V. Galitsyna, Zukhra B. Khasanova, Anton Y. Postnov, Elena I. Yarygina, Tatiana P. Shkurat, Alexander N. Orekhov

**Affiliations:** ^1^15a 3rd Cherepkovskaya Str, National Medical Research Center of Cardiology, Moscow 121552, Russia; ^2^8 Baltiyskaya Str, Institute of General Pathology and Pathophysiology, Moscow 125315, Russia; ^3^K.I. Skryabin Moscow State Academy of Veterinary Medicine and Biotechnology-MVA, Moscow 109472, Russia; ^4^Department of Genetics, 105/42 B. Sadovaya Str, Southern Federal University, Rostov-on-Don 344006, Russia; ^5^Institute for Atherosclerosis Research, Skolkovo Innovative Centre, Moscow 121609, Russia

In response to the letter by Finsterer and Zarrouk-Mahjoub [1] about our article [2], we would like to make several clarifying remarks.

First, the question on the usefulness of any molecule or biochemical variable as a biomarker of some disease is arguable and rather philosophic *per se*. Formally, the conception of evidence-based medicine declares that mechanistic role of any biomarker should be supported by findings from numerous clinical studies and trials [3]. Quite obviously, mitochondrial DNA (mtDNA) variants cannot be regarded at present as estimated and proven biomarkers of predisposition to atherosclerosis, mainly due to insufficient data from unrelated studies, but in any case, they deserve further investigations. In our study, we have demonstrated the direct or reverse association of mitochondrial DNA single nucleotide substitutions (m.652delG, m.3336C>T, m.12315G>A, m.14459G>A, m.15059G>A, m.13513G>A, and m.14846G>A) with carotid subclinical atherosclerosis [2]. Therefore, our data suggest considering mtDNA variants as potential biomarkers for assessing genetic predisposition to disease. In fact, we did not search for specific biomarkers and did not assess interaction of cardiovascular risk factors; limitations of our study were clearly indicated. In the above paper, we have provided in-depth statistical analysis of the association between mtDNA heteroplasmy and cumulative mutational burden and the presence of subclinical carotid atherosclerosis (not manifested plaques in any visualized segment of carotid arteries) or diffuse intima-media thickening of the common carotid artery (cIMT) as phenotypic marker of predisposition to atherosclerosis.

Second, we should note that heteroplasmy levels were measured not in lymphocytes but in leukocytes. It is known that heteroplasmy rates may vary considerably between different tissues. It is true mainly for somatic mtDNA mutations, since during cell division occurs asymmetric distribution of mitochondria, the genome of which contains a mutant allele. The proportion of mutant copies of mtDNA carrying both inherited and somatic mutations may be changed over a lifetime due to unequal separation of mitochondrial genotypes during cytokinesis in dividing cells (vegetative segregation) or in nondividing cells during mtDNA replication. An increase in the degree of heteroplasmy represents some kind of “clonal expansion” of low-level inherited variants, which occurs due to preferential replication of mtDNA carrying certain types of mutations [4]. However, we have earlier shown that there were no significant differences in the level of heteroplasmy of mtDNA mutations between different types of blood cells (monocytes, neutrophils, lymphocytes, and platelets) from the same individual in 71 study participants [5]; so, mutations are not accumulated during differentiation of blood cells but more probably are inherent in the progenitor cells in the bone marrow. Additionally, we have shown that in maternal relatives in 2–4 generations (37 families), in whom genotyping was carried out on heteroplasmic variants m.1555A>G, m.5178C>A, m.3256C>T, m.13513G>A, m.12315G>A, m.14846G>A, and m.15059G>A, the probability of hereditary nature of variants varied from 92% to 100%, but the probability of somatic nature arising in any generation varied from 5% to 19% [5]. Thus, the prevalent variants of the mitochondrial genome are thought to be heritable with a high degree of probability; therefore, they may be met in any tissue. We have also demonstrated mosaic distribution of mtDNA variants in different types of atherosclerotic lesions and overlapping of the profiles of mtDNA mutations in atherosclerotic plaques, blood leukocytes, and buccal epithelial cells [6–10]. The most reliable hypothesis is that the circulating cells with impaired mitochondrial function due to the presence of mutant mtDNA copies would enter into the arterial intimal layer and participate in the processes of atherogenesis. If leukocyte function is inhibited due to the presence of mutations in coding regions of mtDNA, this may lead to local oxidative stress and other pathologic events which could promote atherosclerosis formation [9]. Thus, it can be assumed that mtDNA damage, being a mechanistic biomarker of defective mitochondrial function in leukocytes, can also be regarded as a biomarker for atherosclerosis and consequent clinical manifestations such as CHD [11, 12].

Third, this paper was focused just on statistical evaluation of the significance of mtDNA variants as specific variables for estimation of predisposition to atherosclerosis development. Therefore, such variables as the presence of arterial hypertension, diabetes mellitus, metabolic syndrome, other comorbidities, smoking status, body mass index, family history of CHD and myocardial infarction, arterial blood pressure, total cholesterol, low-density lipoprotein cholesterol (LDL-C), high-density lipoprotein cholesterol (HDL-C), triglycerides, LDL-C to HDL-C ratio, and current medications were taken as covariates. Thus, the analysis allowed considering mtDNA mutations and cumulative mutational burden in stand-alone way, apart from existing conventional risk factors. It should be also mentioned that study participants were recruited from the general population, with a low rate of regular use of medications that could potentially evolve atherosclerosis progression.

Fourth, we absolutely disagree that “high-sensitivity C-reactive protein, the telomere length, lipid oxidation products (MDA-modified collagen, type IV IgM and IgG antibodies), hyperuricemia, TNF-alpha, and IL-15 polymorphism” are “more specific risk factors for atherosclerosis”; the main concern for these metrics is just their low specificity; that is why they are still not either recommended or widely used as the confirmed risk factors of atherosclerosis.

Fifth, for the development of any kind of mitochondrial cytopathy, pathogenic mtDNA variants should reach a very high level of heteroplasmy, even up to homoplasmy, for example, 100% of mutant mtDNA copies. Therefore, one should consider the threshold effect regarding heretoplasmic levels [13, 14]. As the percentage of mutant mtDNA copies may increase with age, the cellular energy capacity will decrease, this in turn affecting the threshold of a minimal cell function [15]. Theoretically, all cells may harbor mutant mtDNA, but phenotype will evidently depend on the percent of mutant allele. Modeling confirmed that there exists an upper threshold level for mutations, beyond which the mitochondrial population collapses with a concomitant decrease in ATP [16], which results in the phenotypic expression of disease [17]. It was estimated that the proportion of mutant DNA (the level of heteroplasmy) in patients should exceed 50% to evolve clinical manifestations like mitochondrial disorders [18]. In our studies, the observed levels of heteroplasmy did not reach the thresholds necessary for the development of mitochondrial disorders, but possibly were sufficient for altering the expression of oxidative phosphorylation complexes, development of mitochondrial dysfunction and accelerated ROS generation, thus promoting atherosclerosis development [19, 20].

In conclusion, we are sure that mutations of mtDNA due to their higher frequency of occurrence and obvious mechanistic role are promising biomarkers for assessing predisposition to atherosclerosis. We are also satisfied with the fact that our publication has gained readers' interest and led to discussion.
